# Lattice dynamics, mechanical stability and electronic structure of Fe-based Heusler semiconductors

**DOI:** 10.1038/s41598-018-37740-y

**Published:** 2019-02-06

**Authors:** Shakeel Ahmad Khandy, Ishtihadah Islam, Dinesh C. Gupta, Rabah Khenata, A. Laref

**Affiliations:** 1 Department of Physics, Islamic University of Science and Techonology, Awantipora, Jammu and Kashmir 192122 India; 2 Department of Physics, Jamia Millia Islamia New Delhi, New Delhi, 110025 India; 3 Condensed Matter Theory Group, School of Studies in Physics, Jiwaji University, Gwalior, 474011 MP India; 4 Laboratoire de Physiéque Quantique, de la Matie’re et de la Modélisation Mathématique (LPQ3M), Université de Mascara, Mascara, 29000 Algeria; 5 Department of Physics, College of Science, King Saud University, Riyadh, Saudi Arabia

## Abstract

The structural and mechanical stability of Fe_2_TaAl and Fe_2_TaGa alloys along with the electronic properties are explored with the help of density functional theory. On applying different approximations, the enhancement of semiconducting gap follows the trend as GGA < mBJ < GGA + U. The maximum forbidden gaps observed by GGA + U method are E_g_ = 1.80 eV for Fe_2_TaAl and 1.30 eV for Fe_2_TaGa. The elastic parameters are simulated to determine the strength and ductile nature of these materials. The phonon calculations determine the dynamical stability of all these materials because of the absence of any negative frequencies. Basic understandings of structural, elastic, mechanical and phonon properties of these alloys are studied first time in this report.

## Introduction

Significant momentum in the study of intermetallic Heusler alloys has increased over the last decade as these systems exhibit numerous extraordinary capabilities in exposing the desired properties, extending from robust spin-polarization, half-metallic magnetism, magnetoresistance, shape memory effect, spin gapless semiconductor to giant magnetocaloric effect, phase transitions and thermoelectric effect^1–7^. The technological applications exploiting these properties have been achieved successfully. Spintronic and thermoelectric applications are the offshoots of half-metallic ferromagnetism (being castoff in spin injectors^8^, spin filters^9^, magnetic tunnel junctions^10^, spin valves^11^, random access memories^12^) and spin gapless attributes to the Seebeck effect useful for thermoelectric devices^13^. Within these dimensions, the materials with compatible lattice structure, high spin polarization and high Curie temperature are anticipated in practical spintronic applications. Magnetoelectronic devices mostly depend on the disproportionate number of majority and minority spin carriers, as exhibited ideally by half-metallic materials i.e. 100% spin polarization at the Fermi level. Such materials display the concoction properties of semiconductor and metal. Additional motive to delegate Heusler alloys in these applications is that these systems have the same crystallographic structure with different functional characteristics and some of them are even very close in electronic structure and composition^14,15^. Since the discovery of the NiMnSb Heusler alloy in 1983^16^, a sequence of experimental as well as theoretical efforts (first principles simulations) were attempted to predict novel semiconductor or half-metallic systems. Among such compounds, transition metal based Heuslers have been widely investigated by material scientists worldwide. Predominantly, the Fe based Heusler structures constitute a vast family with semiconducting or half-metallic band profiles. For example, Fe_2_YSi (Y=Cr, Mn, Fe, Co, Ni) alloys were experimentally synthesized and predicted to be half-metallic alloys^17^. Fe_2_TiAl was reported to have thermoelectric applications^18^. Other materials like, Fe_2_TiSi, Fe_2_TiGe and Fe_2_ZrSi^19^, FeMnSi^20^, FeVRuSi^21^ and many more to report here have been investigated for their magnetic, semiconducting or mechanical properties. Using first-principle calculations, Fe_2_YZ (Y=V, Ti, Nb, Zr, Ta, Hf and Z=Al, Ga, In, Sn, Ge, Si) Heusler compounds with room temperature power factors 4 to 5 times larger than classical thermoelectrics were reported recently by *Bilc et al*.^22^. However, a little information is available on the electronic structure, mechanical stability, phonon dynamics and bonding characteristics of Fe_2_TaAl and Fe_2_TaGa alloys. In addition, the untouched lattice dynamical parameters and phonon properties are necessary to understand the intriguing physical properties and hence in this work, we tried to investigate their structural and mechanical stability, electronic and lattice dynamical properties in detail.

## Results and Discussion

### Structural and mechanical stability

**Figure 1:**
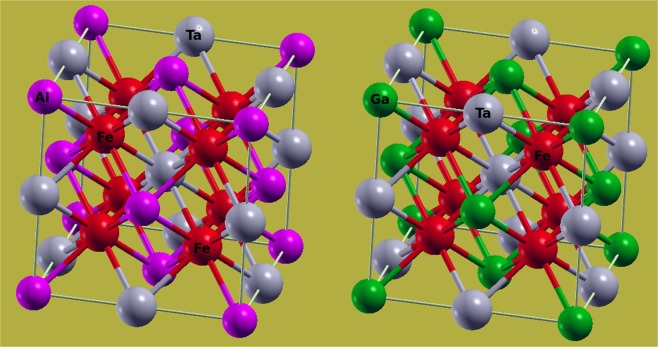
Crystal structure of conventional unit cell for Fe_2_TaAl and Fe_2_TaGa in Fm-3m configuration.

Full-Heusler ternary alloys of Fe_2_TaAl and Fe_2_TaGa type have been found to crystallize in a cubic structure with space group (Fm-3m) as depicted in Fig. 1^22^. The corresponding atomic locations are Fe (1/4, 1/4, 1/4), Ta (1/2, 1/2, 1/2) and Z (0, 0, 0). The ground state structure is determined by geometry optimization via the total energy per unit cell (see Supporting Information) and thereby the equilibrium lattice constants, derivative of bulk modulus, total energy and equilibrium volume are obtained as shown in Table 1. These optimized lattice parameters are fetched out to calculate the ground state properties of these alloys.

**Table 1:** Calculated values of the lattice constant (a_o_), unit cell volume (V_0_), derivative of bulk modulus (B’), ground-state energy (E_0_) and energy gaps (ΔE) of Fe_2_TaAl and Fe_2_TaGa alloys.

Compound	a_o_ (Å)	V_0_ (a.u)	B′	E_0_ (Ry)	ΔE_GGA_ (eV)	ΔE_mBJ_ (eV)	ΔE_GGA+U_ (eV)
Fe_2_TaAl	5.92	350.50	3.95	−36829.20	0.27	0.80	1.80
Fe_2_TaGa	5.85	338.58	5.00	−40231.73	0.02	0.61	1.30

The mechanical stability and elastic response of any crystal system is measured from the elastic constants and thereby the indication of its mechanical properties; e.g Bulk/Young’s/shear moduli, Debye or melting temperature, poisons ratio, brittle/ductile or hardness, etc are derived from them. Macroscopic distortion of a crystal structure is directly related to the elastic constants and is applicable in the evaluation of elastic energies or strains in materials under applied (internal/external/thermal) stresses^23^. These constants are determined by the linear response of a crystal towards the external forces, and are also associated with structural stability, equation of state (EOS), interatomic potential and phonon spectra^24^. Beyond this, the thermal properties including specific heat, thermal expansion, Debye temperature and Gruneisen parameter are exclusively linked to elastic constants. Consequently, the determination of elastic constants is indispensable to characterize a solid crystal. In this work, we have calculated the elastic constants of Fe_2_TaAl and Fe_2_TaGa systems using the generalized gradient approximation (GGA) and are listed in Table 2. For a simple cubic system, the elastic stiffness constants (C_ij_) are reduced to only three independent constants *viz*. C_11_, C_12_, and C_44_. From Table 2, it can be seen that the comprehensive Born criteria; C_12_ < B < C_11_, (C_11_ − C_12_) > 0, (C_11_ + 2C_12_) > 0 and C_44_ > 0 is rigorously followed by the observed lattice constants and hence their mechanical stability is confirmed^25^. Further, to get the information about the different elastic moduli and other derivables, Viogt-Reuss-Hill method is used via the Eqns (1–3)^26–28^,

1 $${G}_{V}=\frac{({C}_{11}-{C}_{12}+3{C}_{44})}{5}\,;\,{G}_{R}=\frac{5({C}_{11}-{C}_{12}){C}_{44}}{4{C}_{44}+3({C}_{11}-{C}_{12})}\,;\,G=\frac{{G}_{V}+{G}_{R}}{2}$$

where, bulk modulus is represented as,

2 $${B}_{V}={B}_{G}=B=\frac{({C}_{11}+2{C}_{12})}{3}$$

and, the Youngs modulus, Poisson’s ratio and anisotropy parameter are defined as

3 $${\rm{Y}}=\frac{9{\rm{BG}}}{3{\rm{B}}+{\rm{G}}}\,;\,\upsilon =\frac{3{\rm{B}}-{\rm{Y}}}{6{\rm{B}}}\,;\,A=\frac{2{{\rm{C}}}_{44}}{({{\rm{C}}}_{11}-{{\rm{C}}}_{12})}$$

**Table 2:** Calculated values of elastic (C_11_, C_12_, C_44_), bulk (B), Shear (G), Young’s (Y) moduli (in GPa), Poisson’s ratio (υ), Zener anisotropy factor (A), B/G ratio, Cauchy’s pressure (C”), and Melting Temperature (*T*_*m*_) in K for Fe_2_TaAl and Fe_2_TaGa alloys.

Parameter	Fe_2_TaAl	Fe_2_TaGa
C_11_	445.56	464.75
C_12_	195.86	224.07
C_44_	125.55	99.89
B	277.72	299.91
G_V_	125.26	108.07
G_R_	125.26	107.17
G	125.26	107.62
Y	326.66	288.36
υ	0.30	0.33
A	1.00	0.82
B/G	2.21	2.78
C”	70.31	124.18
Tm	3186.70 ± 300	3300.13 ± 300

Calculated results for the values of elastic parameters (B, G, Y, A, v, C”, B/G, *etc.)* are summed up in Table 2. The hardness of a material is generally delivered from the observed values of bulk modulus (B) and shear modulus (G). Indisputably, when a stress is applied to any system, the opposition offered at the critical point before which the system is fractured refers to the hardness of that material^29^. Concurrently, the ductile and brittle character of materials is directly linked to the critical value (1.75) of B/G ratio. If B/G is less than the critical value, then the material is ductile otherwise it is said to be brittle^30^. The covalent materials (e.g. Diamond) in principle are relatively hard and obviously brittle with a smaller Pugh ratio. The strong covalent bonds in such materials certainly produce a significant resistance resulting in a quite high hardness. Conversely, ductile materials with a high Pugh’s ratio are characterized by metallic bonding and low hardness. Consequently, the observed values of B, G and B/G clearly determine the Fe_2_TaAl alloy to be much harder and ductile from Fe_2_TaGa compound. This claim is also supported by the (C” = *C*_12_–*C*_44_) values because its positive value defines the ductile nature of the present alloys and if its value is negative, then the system is said to be brittle in nature^31^. Positive Cauchy pressure also specify the presence of metallic bonding in a material while as the negative value demonstrates the directional (covalent) and angular bonds. In the present set of calculations, Cauchy pressure predicted for both the Heusler alloys are positive which reflects their metallic character. Poisson’s ratio and its critical value; 0 < υ < 0.5 simply defines the plasticity of a crystal. Its small value reflects the maximum plastic character and conversely, the material is elastic in nature^32^. So, the elastic nature of these alloys can be observed from the υ values mentioned in Table 2. At the same time, the value of anisotropic factor (A) for a perfectly isotropic system is equal to 1 and its value below or above unity proposes the anisotropic character of a compound. Therefore, the Fe_2_TaAl alloy is purely isotropic but the Fe_2_TaGa alloy is anisotropic in nature^31^.

The thermodynamic behavior of the Fe-based Heusler systems has been described by the calculation of melting temperature (T_m_). Using the empirical equation (Eqn. 4) given below, the melting temperature of the present Heusler systems has been premeditated^33,34^.

4 $${T}_{m}(K)=[553(K)+(5.911){c}_{11}]GPa\pm 300K$$

The large values of *T*_*m*_ as seen from the Table 2 imply the strength of the present materials against the temperature and this hints about the retention of their ground state crystal structures at raised temperatures.

### Semiconducting gap and electronic structure

**Figure 2:**
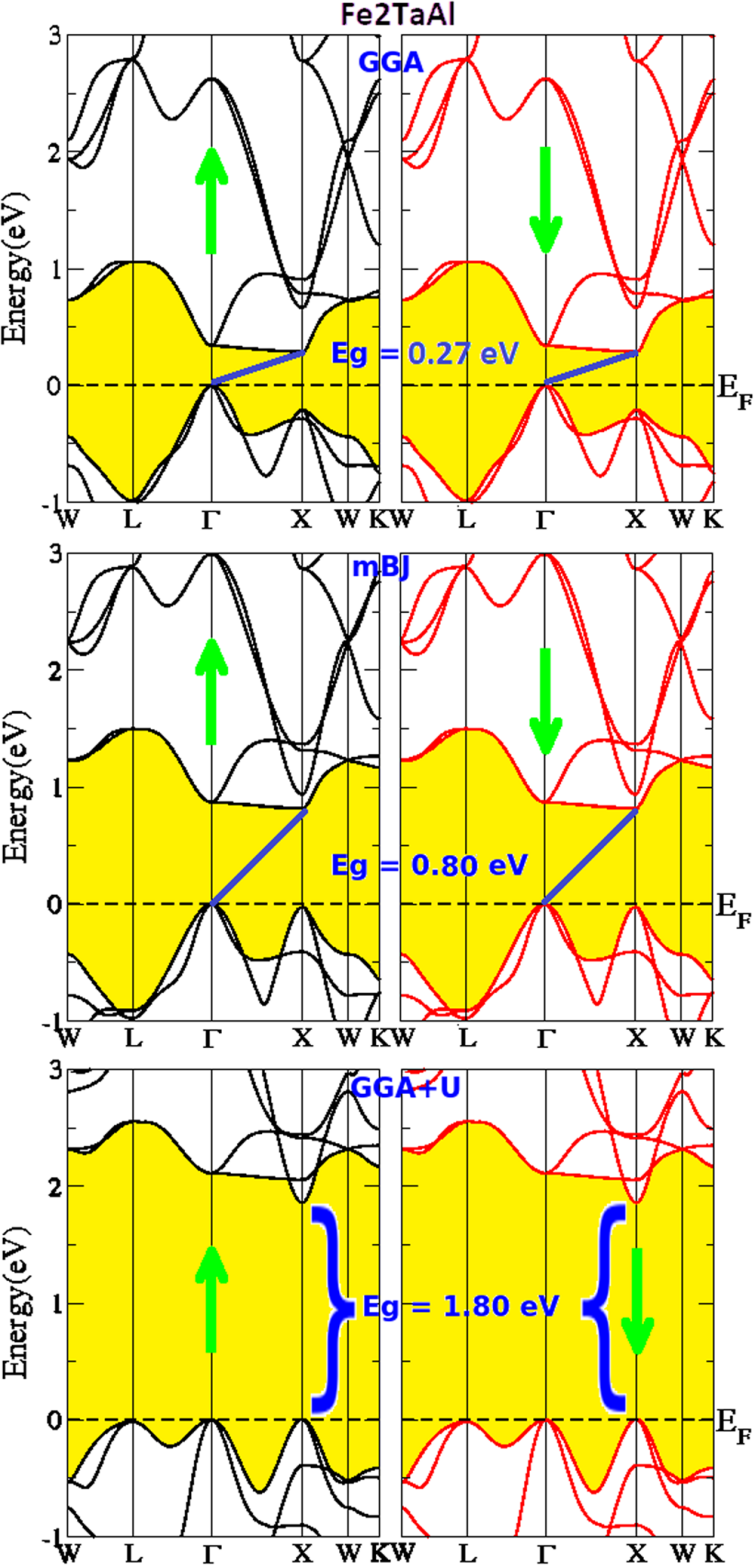
Calculated band structures of Fe_2_TaAl by GGA, mBJ and GGA + U methods.

**Figure 3:**
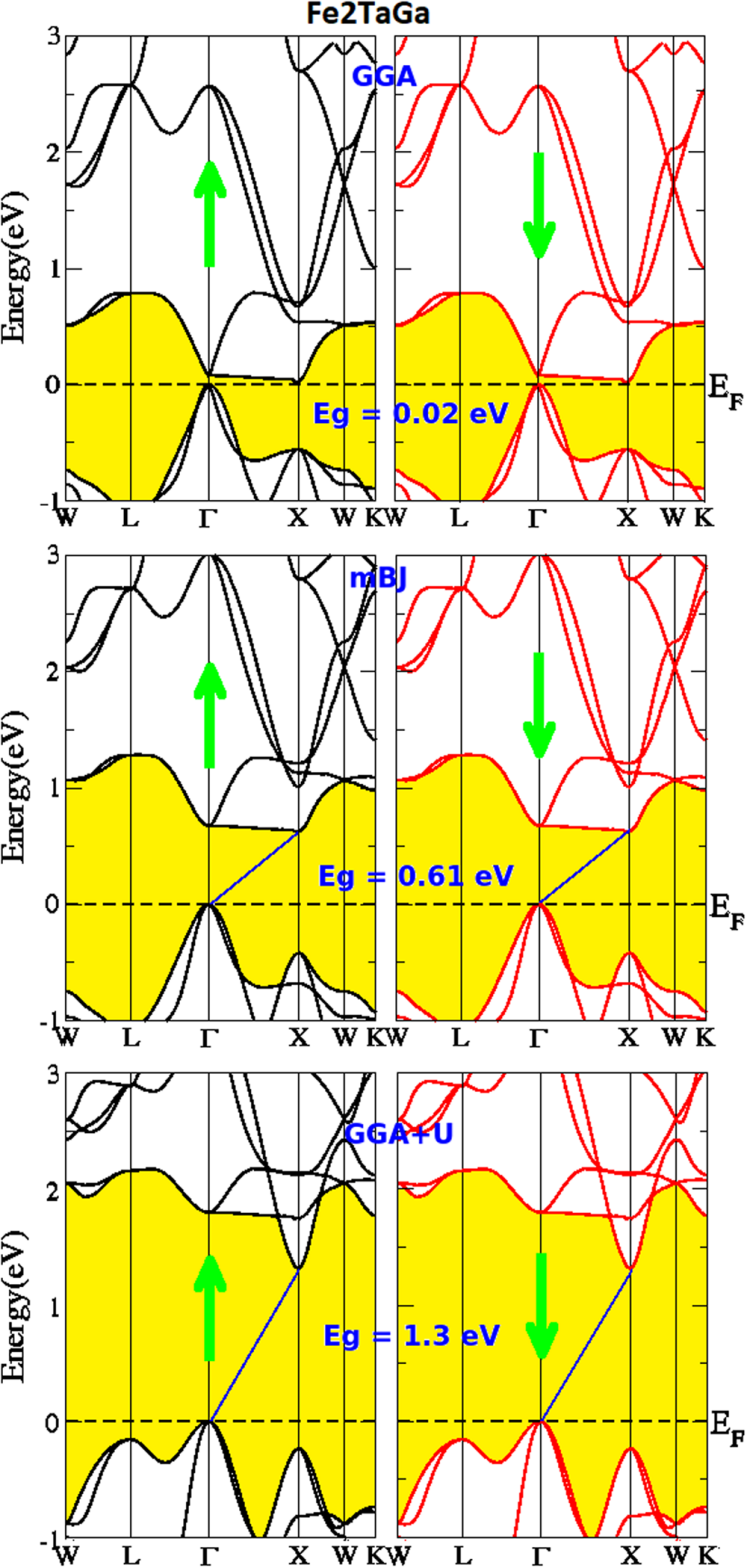
Calculated band structures of Fe_2_TaGa by GGA, mBJ and GGA + U methods.

Since both these alloys have 24 valence electrons (Z_t_) in their equilibrium structures, therefore, Slater-Pauling rule^34^; (M_t_ = Z_t_ − 24) comprises the zero-spin magnetic moment for both these materials. Their non-magnetic character has earlier been reported in ref.^22^. The groundwork of structural optimization yields the equilibrium lattice constant, and the same is utilized to calculate the electronic band profile of Fe_2_TaAl and Fe_2_TaGa Heusler materials. Three different schemes; generalized gradient approximation (GGA), onsite Hubbard approximation (GGA + U) and modified Beckhe Johnson (mBJ) schemes have been employed and the spin polarized band profiles are displayed in Figs 2 and 3. The GGA and mBJ calculated energy gaps are very small as compared to GGA + U calculations. However, GGA + U clearly widens the gap between valence and conduction bands in both the compounds. From GGA + U calculations, Fe_2_TaAl is observed to be a direct band gap material with Eg = 1.80 eV, while as Fe_2_TaGa is observed to be indirect band gap semiconductor with the corresponding energy gap of 1.30 eV. In the latter case, the valence band maximum (VBM) occurs at the Γ symmetry point and the conduction band minimum (CBM) occurs at the X symmetry point in its Brillouin zone. However, GGA and mBJ methods reveal both these materials to be the p-type indirect band gap semiconductors.

**Figure 4:**
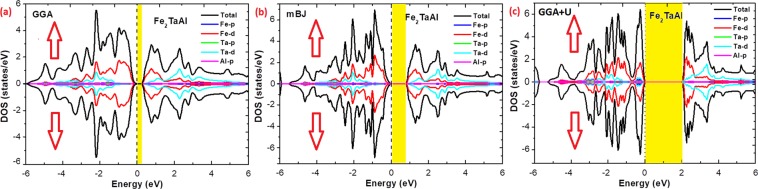
Observed total densities of states (DOS) and partial densities of states (pDOS) of Fe_2_TaAl compound calculated by GGA, mBJ and GGA + U schemes.

**Figure 5:**
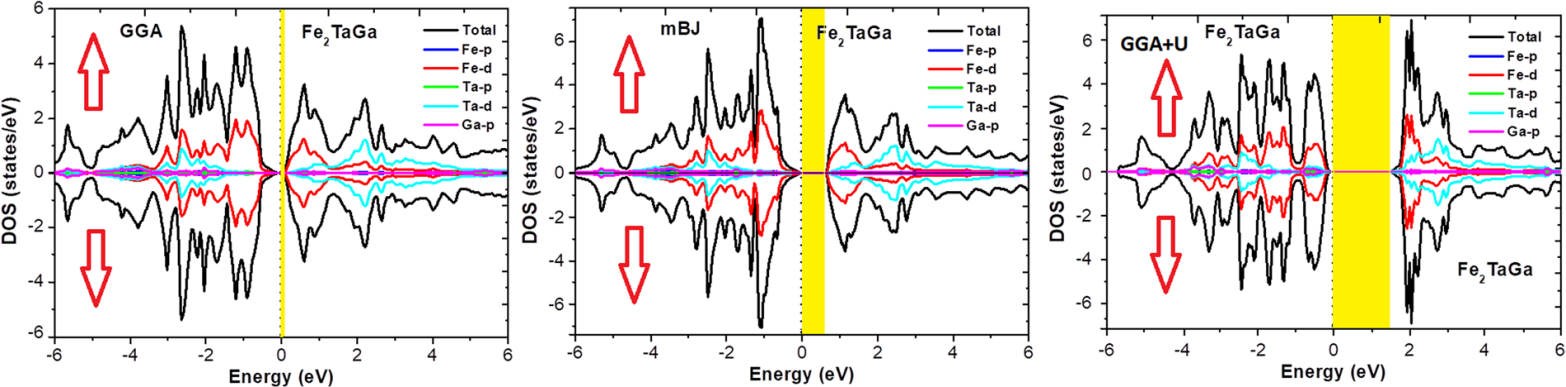
Observed total densities of states (DOS) and partial densities of states (pDOS) of Fe_2_TaGa compound calculated by GGA, mBJ and GGA + U schemes.

Further, the spin polarized total density of states (TDOS) depicted in Fig. 4 and partial density of states (PDOS) shown in Fig. 5 for the present materials at their equilibrium lattice constants are discussed. Since, the total DOS in up and down spin channels are same (cancel each other), therefore the non-magnetic character of these alloys can be estimated. Also, the pDOS of these materials by GGA underestimates the band structure and therefore small or negligible gaps are observed by this method. Consequently, the onsite Hubbard correction (described in section 4 for Fe-d states) widens the band gap comparatively. The Fe-d and Ta-d states are mostly populated around the Fermi level with a maximum contribution towards the total DOS and consequently, the corresponding bonding-antibonding states control the energy gap formation. At the same time group IV atomic states are less active around the Fermi level in these materials. Thus, the observed band gap in these alloys is due to the typical *d-d* hybridization between the valence states of Fe and Ta atoms and the same has been explained elsewhere for similar materials like Co_2_TaAl and Co_2_TaGa^3,28^. Hence, from the observed band profiles and densities of state plots, both the compounds are found to be p-type indirect band gap semiconductors. Present calculations unlock the potential application of these alloys in semiconductor and energy harvesting technologies.

### Phonon properties and Cohesive Energies

**Figure 6:**
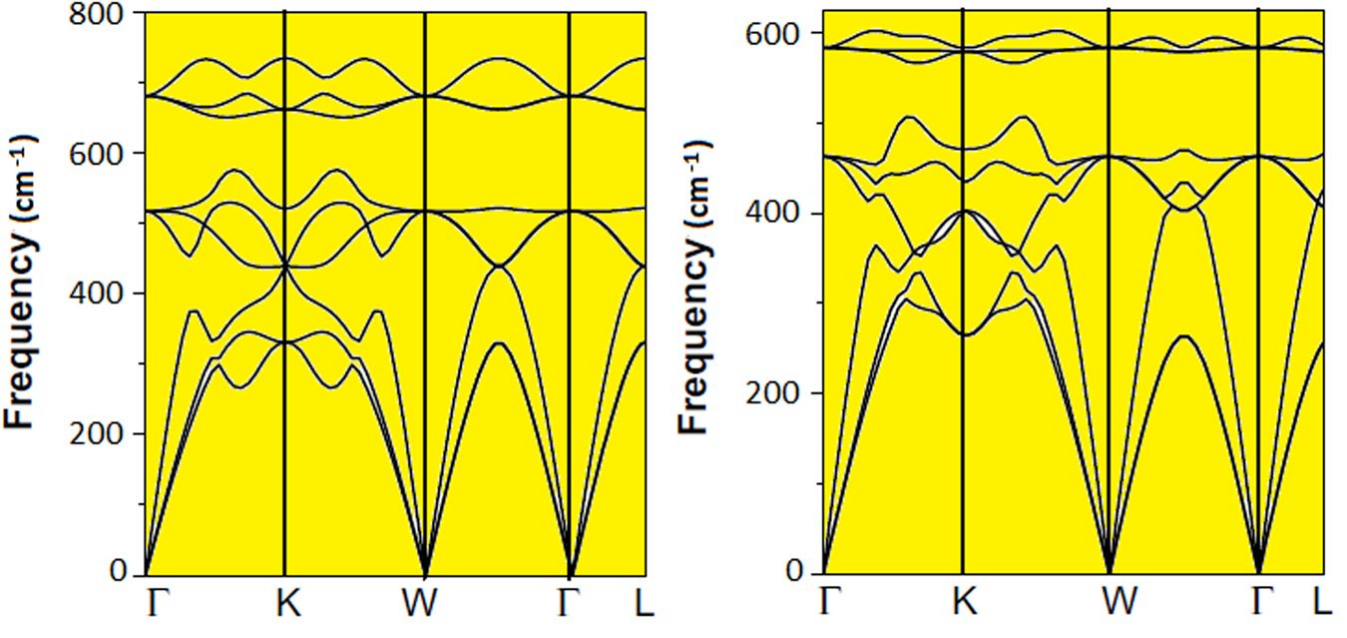
Phonon dispersion for Fe_2_TaAl and Fe_2_TaGa alloys.

Phonon dispersions are interesting phenomenon to understand the dynamical stability of a crystal system above and beyond knowing their thermal behavior, superconductivity, Raman and thermal spectroscopy^35–37^. Figure 6(a,b) shows the phonon dispersion curves (PDCs) of these alloys obtained using GGA scheme. The four atoms of Fe_2_TaAl and Fe_2_TaGa system in its unit cell give rise to three acoustical and nine optical modes constituting a total of 12 phonon branches. But, in different directions, this number is reduced due to degeneracy. The optical modes obtained for Fe_2_TaAl are at around 510.19 cm^−1^ and 680.22 cm^−1^ and for Fe_2_TaGa are around 470.29 cm^−1^, 590.63 cm^−1^ at zone center Г and W points.

It can also be seen from Fig. 4(a,b) that the longitudinal optical-transverse optical (LO-TO) splitting in our materials is almost nil at Г and W symmetry points. The difference of LO and TO (*ω*_*LO*_ − *ω*_*TO*_) is called a reststrahlen band, which estimates the number of reflected electromagnetic waves. In our calculation ${\omega }_{LO}^{2}-{\omega }_{TO}^{2}\approx 0$ at Г and W points, hence exhibit higher phonon scattering ability. Such phenomenon can obviously enhance the thermoelectric response of these materials by decreasing thermal conductivity. The dynamical stability is confirmed by the absence of imaginary frequency in all high symmetry direction for both the investigated compounds.

Since, the stability of both these materials has been confirmed via Convex Hull analysis with −303.3 MeV for Fe_2_TaAl and −64.3 MeV for Fe_2_TaGa a**s** the energy above the hull which is equal to the energy of formation of the compounds of interest from the phases that would be stable if the compound did not exist^22^. Therefore, our phonon results strengthen the stability criteria of these alloys. Moreover, we have calculated the cohesive energies (*E*_*coh*_) of these compounds using the following equation:

$${E}_{Coh}=(2{E}_{Fe}^{iso}+{E}_{Ta}^{iso}+{E}_{Al/Ga}^{iso})-{E}_{F{e}_{2}TaAl/Ga}^{Total}$$

where ${E}_{Fe}^{iso}$, ${E}_{Ta}^{iso}$ and ${E}_{Al/Ga}^{iso}$ are the isolated atomic energies of the Fe, Ta and Al/Ga atoms, respectively, and ${E}_{F{e}_{2}TaAl/Ga}^{Total}$, is the total energy of Fe_2_TaAl and Fe_2_TaGa per formula unit. The measurement of strength of the binding force between the constituent atoms in a solid structure is confirmed from cohesive energy its positive value indicates the stability of the material. The calculated values of the cohesive energy are 22.71 eV for Fe_2_TaAl compound and 20.67 eV for Fe_2_TaGa alloy. These observed values are significant and comparable to Co_2_TaSi (21.76 eV), Co_2_TaGe (19.18 eV)^38^ and Hf_2_Val (21.56 eV)^39^; and this specifies the large value of chemical bond energy. Therefore, the stability of the present materials is confirmed from the mechanical stability criteria, cohesive energy and phonon dispersion results.

## Conclusion

In this study, the electronic and mechanical properties of cubic Fe_2_TaAl and Fe_2_TaGa alloys have been examined using the density functional theory. Calculations of the electronic structure shows the indirect band gaps along Γ-L symmetry for all these materials. These compounds directly fall under the Slater Pauling rule that depicts their semiconducting behavior. Thus, we have confirmed from our studies that these full-Heusler alloys are narrow band gap semiconductors with 0.80 eV for Fe_2_TaAl and 0.61 eV for Fe_2_TaGa. In relation to electronic structure, the mechanical properties have been calculated. A theoretical study predicts the hardness and ductile nature of these materials and their ductility increases from Al > Ga and are potentially the possible hard semiconductor materials. In this study, the electronic, phonon and mechanical properties of cubic Fe_2_TaAl and Fe_2_TaGa alloys have been examined using the density functional theory. The calculation of lattice vibrations from DFPT gives the phonon dispersion. The coupling of optical and acoustic mode is an interesting phenomenon that we have observed in this study with zero LO-TO splitting at Г and W points of symmetry. The zero value of reststrahlen band is responsible for higher value of phonon scattering and reduced the lattice thermal conductivity.

## Methodology

The investigation of structural, elastic, electronic and phonon properties of the Fe_2_TaAl and Fe_2_TaGa compounds, first-principles calculations using the full potential linearized augmented plane-wave method (FPLAPW)^40^ as executed in the WIEN2k Package^41^. This quantum mechanical code can precisely simulate the ground state structure, band gaps, dielectric properties, magnetic properties, and so on. The generalized gradient approximation (GGA)^42^, mBJ^43^ and onsite Hubbard correction (GGA + U)^44^, were adopted for the exchange-correlation potentials. In GGA + U calculations, we have used the U-J = 4.0 eV for Fe-d electrons with J = 0 according to the Dudarav’s method^45^. A dense k-point mesh of 10 × 10 × 10 was used in the Brillouin zone integrations. The basis functions are expanded up to R_MT_K_max_ = 7, where RMT is the smallest atomic radius in the unit cell and K_max_ refers to the magnitude of the largest k vector in the plane wave expansion. The total energy convergence tolerance for the calculations was selected within 1 × 10^−6^ eV. Furthermore, the elastic properties were calculated by cubic elastic code^46^ and from these elastic constants other mechanical parameters were determined to discuss the mechanical response of these materials. DFT has proven to be one of the successful methods to predict the stability as well as ground state properties of the materials theoretically^47,48^. The lattice dynamical properties were analyzed in term of phonon frequencies as a second-order derivative of the total energy with respect to atomic displacements within the frame work of DFPT^49^ by using the Quantum Espresso package^50^.

## Supplementary information

Lattice dynamics, mechanical stability and electronic structure of Fe-based Heusler semiconductors
