# Supplementary material for: Lattice dynamics, mechanical stability and electronic structure of Fe-based Heusler semiconductors
Source: Sci Rep. 2019 Feb 6;9:1475. doi: 10.1038/s41598-018-37740-y (PMC6365636; doi:10.1038/s41598-018-37740-y)
Supplement: Supplementary file 1 — Lattice dynamics, mechanical stability and electronic structure of Fe-based Heusler semiconductors [file 41598_2018_37740_MOESM1_ESM.docx]

**Lattice dynamics, mechanical stability and electronic structure of Fe-based Heusler semiconductors**

Shakeel Ahmad Khandy^a,*^, Ishtihadah Islam^b^, Dinesh C Gupta^c^, Rabah Khenata^d^, and A. Laref^e^

^a^Department of Physics, Islamic University of Science and Techonology, Awantipora, Jammu and Kashmir-192122- India

^b^Department of Physics, Jamia Millia Islamia New Delhi –110025, India

^c^Condensed Matter Theory Group, School of Studies in Physics, Jiwaji University, Gwalior-474011 (MP), India

^d^Laboratoire de Physique Quantique, de la Matie`re et de la Mode´lisation Mathe´matique (LPQ3M), Universite´ de Mascara, Mascara 29000, Algeria

^e^Department of Physics, College of Science, King Saud University, Riyadh – Saudi Arabia

Email:[shakeelkhandy11@gmail.com](mailto:shakeelkhandy11@gmail.com)





The volume versus energy plots of the Fe-based alloys obtained via Birch-Murnaghn’s equation in Fm-3m configuration
